# Specifying the target difference in the primary outcome for a randomised controlled trial: guidance for researchers

**DOI:** 10.1186/s13063-014-0526-8

**Published:** 2015-01-15

**Authors:** Jonathan A Cook, Jenni Hislop, Douglas G Altman, Peter Fayers, Andrew H Briggs, Craig R Ramsay, John D Norrie, Ian M Harvey, Brian Buckley, Dean Fergusson, Ian Ford, Luke D Vale, for the DELTA group

**Affiliations:** Centre for Statistics in Medicine, Nuffield Department of Orthopaedics, Rheumatology and Musculoskeletal Sciences, University of Oxford, Botnar Research Centre, Nuffield Orthopaedic Centre, Windmill Road, Oxford, OX3 7LD UK; Health Services Research Unit, University of Aberdeen, Health Sciences Building, Foresthill, Aberdeen, AB25 2ZD UK; Institute of Health and Society, Newcastle University, The Baddiley-Clark Building, Richardson Road, Newcastle upon Tyne, NE2 4AX UK; Population Health, University of Aberdeen, Polwarth Building, Foresterhill, Aberdeen, AB25 2ZD UK; Department of Cancer Research and Molecular, Norwegian University of Science and Technology, Mailbox 8905, Trondheim, N-7491 Norway; Health Economics and Health Technology Assessment, University of Glasgow, 1 Lilybank Gardens, Glasgow, G12 8RZ UK; Centre for Healthcare Randomised Trials (CHaRT), University of Aberdeen, Health Sciences Building, Aberdeen, AB25 2ZD UK; Faculty of Medicine and Health Sciences, University of East Anglia, Elizabeth Fry Building, Norwich Research Park, Norwich, NR4 7TJ UK; National University of Ireland, University Road, Galway, Ireland; Clinical Epidemiology Program, Ottawa Hospital Research Institute, 725 Parkdale Avenue, Ottawa, ON K1Y 4E9 Canada; Robertson Centre for Biostatistics, University of Glasgow, Boyd Orr Building, University Avenue, Glasgow, G12 8QQ UK

**Keywords:** Target difference, clinically important difference, sample size, randomised controlled trial, guidance

## Abstract

**Background:**

Central to the design of a randomised controlled trial is the calculation of the number of participants needed. This is typically achieved by specifying a target difference and calculating the corresponding sample size, which provides reassurance that the trial will have the required statistical power (at the planned statistical significance level) to identify whether a difference of a particular magnitude exists. Beyond pure statistical or scientific concerns, it is ethically imperative that an appropriate number of participants should be recruited. Despite the critical role of the target difference for the primary outcome in the design of randomised controlled trials, its determination has received surprisingly little attention. This article provides guidance on the specification of the target difference for the primary outcome in a sample size calculation for a two parallel group randomised controlled trial with a superiority question.

**Methods:**

This work was part of the DELTA (Difference ELicitation in TriAls) project. Draft guidance was developed by the project steering and advisory groups utilising the results of the systematic review and surveys. Findings were circulated and presented to members of the combined group at a face-to-face meeting, along with a proposed outline of the guidance document structure, containing recommendations and reporting items for a trial protocol and report. The guidance and was subsequently drafted and circulated for further comment before finalisation.

**Results:**

Guidance on specification of a target difference in the primary outcome for a two group parallel randomised controlled trial was produced. Additionally, a list of reporting items for protocols and trial reports was generated.

**Conclusions:**

Specification of the target difference for the primary outcome is a key component of a randomized controlled trial sample size calculation. There is a need for better justification of the target difference and reporting of its specification.

**Electronic supplementary material:**

The online version of this article (doi:10.1186/s13063-014-0526-8) contains supplementary material, which is available to authorized users.

## Background

Well-conducted randomised controlled trials (RCTs) are widely viewed as providing the optimal evidence on the relative performance of competing healthcare interventions [1,2]. However, simply detecting any statistical difference in the effectiveness of interventions may not be sufficient or useful; if the interventions differ to a degree or in a manner that is of little consequence in patient, clinical or economic (or other meaningful) terms, then the interventions might be considered not to be different. If RCTs are to produce useful information that can help patients, clinicians and planners make decisions about health care, it is essential that they are designed to achieve this. This is typically achieved by specifying a target difference for a primary outcome as part of a sample size calculation, which provides reassurance that the trial will have the specified statistical power to identify whether a difference of a particular magnitude exists. Beyond purely statistical or scientific concerns, the sample size calculation has financial and ethical implications. Failing to recruit sufficient participants to be able to confidently detect a relevant difference between interventions may be viewed as an inefficient use of finite research resources, while recruiting substantially more than are needed risks exposing participants to unnecessary experimentation [3].

Given these considerations, determining an appropriate sample size is of critical importance. Surprisingly, little practical advice is available on specifying the target difference of the chosen primary outcome, which as noted above is a key component of the sample size calculation. A comprehensive systematic review of the literature identified methods for determining the target difference that are available and surveys have shown these methods are in use [4,5]. Nevertheless, uncertainty regarding the magnitude of the target difference when designing the trial will lead to uncertainty regarding the interpretation of the results, even when the trial is otherwise successfully conducted [6,7].

This article aims to provide practical guidance primarily for researchers involved in determining the sample size for an RCT and, in particular, the specification of the target difference in the primary outcome. It is also relevant to those who are involved in commissioning and publishing such studies. We provide guidance on the choice of the primary outcome, specification of the target difference and a brief summary of available methods that can be used to inform its specification and reporting. Additionally, two sets of reporting items, one for a trial protocol and the other a report of the trial findings in a peer reviewed biomedical journal, are also proposed and examples provided. A comprehensive systematic review and discussion of the individual methods for specifying a target difference has been reported elsewhere [4,5]. The focus of this guidance is upon what might be termed the conventional, or standard, approach to an RCT sample size calculation: a standalone trial utilising the conventional statistical framework for sample size calculation and primarily for superiority trials (those where the difference to be detected is specified). The key issues considered are relevant to other RCT designs and analysis approaches though implementation may differ. We note that the conventional approach to sample size calculation is not without its limitations and alternatives have been proposed [8], nevertheless it continues to be the most widely adopted approach [1,9].

The conventional approach to the sample size calculation for a two parallel group RCT is as follows:

1. The RCT is conceived as a standalone definitive study (a study that is designed to provide a meaningful answer on its own);
2. It addresses a superiority question evaluating evidence of a difference (in either direction);
3. Adoption of a two parallel-group RCT design (typically 1:1 allocation);
4. Application of the Neyman-Pearson framework to calculate the sample size [2,10-12]. This requires specification of: the primary outcome for which the required sample size is to be calculated; the target difference (specification varies according to outcome type); statistical parameters (significance level and power) and other component(s) of the sample size calculation (such as standard deviation (SD)).

## Methods

### Development of the guidance

This work was part of the DELTA (Difference ELicitation in TriAls) project, a study on target differences commissioned by the Medical Research Council/National Institute for Health Research Methodology Research Panel (MRC/NIHR) in the United Kingdom. It comprised three interlinking components: a comprehensive systematic review of methods for specifying the target difference, two surveys of current practice amongst clinical trialists and generation of structured guidance. This article is an abridged version of this guidance and other components of the project which have been reported in full elsewhere [4]. DELTA was undertaken by a collaborative group in which the majority of members have extensive experience of the design and conduct of RCTs (both as investigators and as independent committee members) and have conducted methodological research related to RCTs (such as quality-of-life measurement, statistical methodology, reporting, surgical trials and economic evaluation). The draft guidance was developed by the project steering and advisory groups utilising the results of the systematic review and surveys. Findings were circulated and presented to members of the combined group at a face-to-face meeting, along with a proposed outline of the guidance document structure and a list of recommendations and reporting items for a trial protocol and report. Both the structure and main recommendations were agreed at this meeting. The guidance was subsequently drafted and circulated for further comment before finalisation. No ethical approval was needed for this research.

### Scope of the guidance

This guidance is based upon the conventional approach to a sample size calculation, though it should be applicable to most RCTs [1,9]. However, other approaches, for example trials with an explicitly Bayesian analysis framework, will require adaptation of the reporting items. It focuses upon guidance for a trial with a ‘superiority’ question; one which seeks evidence of a difference between intervention groups. Although this guidance is primarily aimed at researchers, it is also relevant for publishers, funders and commissioners of research.

## Results

Abridged guidance is given below.

### Choosing the primary outcome

In the conventional approach to the sample size calculation for an RCT, a single outcome is usually chosen to be the primary measure upon which the sample size calculation is based (in some cases more than one primary outcome may be appropriate) [2,10,13]. The specification of a primary outcome performs a number of functions in terms of trial design, but it is clearly a pragmatic simplification to aid the design, interpretation and use of RCT findings. Through the corresponding sample size calculation and specification of the target difference, it clarifies what the study aims to identify, and the statistical power and precision with which this can be achieved. Stating the primary outcome in the study protocol also helps prevents undue over-interpretation arising from testing multiple outcomes and selective outcome reporting bias, whereby authors report only statistically significant (on possibly clinically irrelevant) outcomes or change the primary focus of the study to match a statistically significance finding. Additionally, it helps clarify the initial basis upon which to judge the study findings. This is particularly important in presence of a ‘negative’ result, where the result does not meet the criteria for statistical significance (typically 5%). In all cases, focus should be upon the confidence interval as well as the point estimate, where a justifiable target difference can guide the interpretation. However, such justification of the target difference is often lacking in trial reports [1,6]. Calculating (or reverse engineering) the magnitude of a difference that can be detected at conventional levels of statistical significance and power (typically two-sided 5% and 80%, respectively), given a sample size which is believed to feasible, is often performed in practice for a selection of key outcomes before determining the primary outcome. Nevertheless, it is important to report the final sample size calculation, including the chosen primary outcome, the target difference and any justification of the value chosen, in as robust and transparent a fashion as possible to allow others to judge the basis of the calculation.

### Specifying the target difference

The specification of the target difference in an RCT sample size calculation has received surprisingly little discussion in the literature. For a superiority trial, it is the difference in the primary outcome value that the study is designed to detect reliably [2,10,13]. There are two main bases for specifying the target difference: a difference considered to be ‘important’ (for example, by a stakeholder group such as health professionals or patients), and a ‘realistic difference’ based upon current evidence (for example, seeking the best available estimates in the literature through some form of knowledge synthesis).

It has been argued that a target difference should always meet both of these criteria [14]. The desire to be able to consider an (clinically) important difference can be viewed as a middle ground between ignoring the consequences of the treatment decision and a full assessment of the benefits, harms and costs of an intervention against the alternatives, which seeks to ensure that any harms and costs are incurred for a good reason. Focusing on a benefit (or harm) of the most important outcome is a natural and intuitive, if imperfect, way to guide a decision. A large body of literature exists on defining a clinically important difference, though not in the context of an RCT sample size calculation [15-17]. The most common general approach is the minimal clinically important difference (MCID). This has been defined as ‘the smallest difference …. which patients perceive as beneficial and which would mandate, in the absence of troublesome side effects and excessive cost, a change in the patient’s management’, or more simply as ‘minimum difference that is important to a patient’ [17]. Many variants on this basic approach exist [18,19]. In the context of specifying a target difference for a typical two parallel-group trial, the focus is on a difference at the group level, between two groups of different participants. This contrasts with the vast majority of the MCID (and related) literature, which focuses overwhelmingly upon within-patient change and whether an important difference can be said to have occurred [15-17]. An alternative approach is to consider all relevant issues, including the consequences of decision-making, whereby a difference of any magnitude can be viewed as important and therefore a study’s size (and implicitly the target difference) is determined by reference to resource implications [20,21]. Whatever definition is used, estimation of an important difference is not without its challenges and limitations [22,23].

The other main basis for a target difference is to specify a realistic difference; there is, for example, little point in setting as the target difference one that is so large that it cannot plausibly exist. If a systematic review of RCTs on the research question is available, it can be used to specify what difference is supported by current evidence. In essence, a realistic difference makes no claim regarding its clinical importance or otherwise. However, where a realistic difference is used, consideration of the importance of the difference is needed if the study findings are intended to inform clinical, patient or policy decisions. For some outcomes, the importance may be very clear (for example, mortality), whereas for others (especially quality of life and surrogate outcomes) further explanation is needed. Recruitment, study management and finance will naturally come into play when determining the sample size of a study. However, such considerations do not negate concerns about what is a realistic and/or important difference.

For a superiority trial it is generally accepted that the target difference should be a clinically important difference [2,10-12] or ‘at least as large as the MCID [minimum clinically important difference]’ [24]. The target difference in a conventional sample size calculation is not the minimum difference that can be statistically detected; statistical significance alone is not a sufficient consideration for attributing importance to a difference [2,12].

The target difference is specified differently depending upon the type of primary outcome. For a continuous outcome, this target difference on either the original or standardised scale is often referred to as the ‘effect size’. Strictly speaking, this value alone does not fully (uniquely) specify the target difference; the assumed variability of the outcome (standard deviation) is also needed to convert the effect size between the original and standardised scales. For a binary outcome, the target difference will be conditional on the control group event proportion. To uniquely specify the sample size, the target difference and the control group event proportion are needed, which together imply a unique pair of absolute and relative target differences. Similarly, survival outcomes require the control group proportion or survival distribution and length of follow-up period to be stated, in addition to the target difference. This is necessary as the sample size required is sensitive to both the absolute level and the relative difference. Despite this, it is not uncommon for only one or the other to be specifically stated in trial reports.

Seven methods for specifying the target difference have been identified [4] which can be used to inform the choice of target difference: anchor, distribution, health economic, opinion-seeking, pilot study, review of the evidence base and standardised effect size (see Table 1 for a brief summary and elsewhere for a summary of the literature assessment of the use of each method [5]).

**Table 1:** **Methods for specifying an important and/or realistic difference** [5]

**Name**	**Description**	**Difference specified**
*Anchor*	The outcome of interest can be ‘anchored’ by using either a patient’s or health professional’s judgement to define an important difference. This may be achieved by comparing a patient’s health before and after treatment, and then linking this change to participants who showed improvement and/or deterioration using a more familiar outcome (for which either patients or health professionals more readily agree on what amount of change constitutes an important difference). An outcome can be anchored to another which more is known about. Contrasts between patients (such as individuals with varying severity of a disease) can also be used to determine a meaningful difference.	Important
*Distribution*	Approaches that determine a value based upon distributional variation. A common approach is to use a value that is larger than the inherent imprecision in the measurement and therefore likely to represent a minimal level for a noticeable difference.	Important
*Health economic*	Approaches that use principles of economic evaluation. These typically include both resource cost and health outcomes, and define a threshold value for the cost of a unit of health effect that a decision-maker is willing to pay, to estimate the overall net benefit of treatment. The net benefit can be analysed in a frequentist framework or take the form of a (typically Bayesian) decision-theoretic value of information analysis. Due to difficulties in implementing a value of information analysis simpler heuristic frameworks, also based on the principles of economic evaluation, have been proposed.	Important
*Opinion-seeking*	The target difference can be based on opinions elicited from health professionals, patients or others. Possible approaches include forming a panel of experts, surveying the membership of a professional or patient body or interviewing individuals. This elicitation process can be explicitly framed within a trial context.	Important and/or realistic
*Pilot study*	A pilot (or preliminary) study may be carried out where there is little evidence, or even experience, to guide expectations and determine an appropriate target difference for the trial. In a similar manner, a phase two study could be used to inform a phase three study, though this would need to take account of methodological differences (such as inclusion criteria and outcomes), which should be reflected in the target difference.	Realistic
*Review of evidence base*	The target difference can be derived using current evidence on the research question. Ideally, this would be from a systematic review or meta-analysis of RCTs. In the absence of randomised evidence, evidence from observational studies could be used in a similar manner. An alternative approach is to undertake a review of studies in which an important difference was determined.	Important and/or realistic
*Standardised effect size*	The magnitude of the effect on a standardised scale defines the value of the difference. For a continuous outcome, the standardised difference (most commonly expressed as Cohen’s d effect size) can be used. Cohen’s cutoff values of 0.2, 0.5 and 0.8 for small, medium and large effects, respectively, are often used. Thus a medium effect corresponds simply to a change in the outcome of 0.5 SDs. Binary or survival (time-to-event) outcome metrics (such as an odds, risk or hazard ratio) can be utilised in a similar manner, though no widely recognised cutoff values exist. Cohen’s cutoff values approximate odds ratios of 1.44, 2.48 and 4.27, respectively. Corresponding risk ratio values vary according to the control group event proportion.	Important

### Reporting the sample size calculation and target difference

The assumptions made in the sample size calculation should be clearly specified. All inputs should be clearly stated so that the calculation can be replicated. It is recommended that trial protocols clearly and fully state the sample size calculations, including where the approach taken differs from the conventional approach (for example, the adoption of a Bayesian framework instead of a frequentist approach), statistical parameters and the target difference, with justification for the choice of values. Due to space restrictions in many publications the main trial paper is likely to contain less detail. A minimum set of items for the main trial results paper along with full specification in the trial protocol is recommended below in Table 2. These are more extensive lists of reporting items building upon the Consolidated Standards for Reporting Trials (CONSORT) including the 2010 version) and Standard Protocol Items: Recommendations for Interventional Trials (SPIRIT) statements, which provide guidance on reporting the sample size calculation, but not explicitly how to report the target difference and its justification [25-27] Examples for the three most common outcome types are provided in Table 3.

**Table 2:** **Reporting items for the protocol and report of a two parallel group superiority trial**

**Item no.**	**Item description**	**Protocol**	**Report**
*1*	State any divergence from the conventional approach.	√	√
*2*	State the primary outcome (and any other outcome which the study sample size calculation is based upon), or state why there is not one.	√	√
*3*	Reference the formula and/or simulation approach if the standard binary, continuous or survival outcome formulas are not used [2,11]. The primary analysis should be stated in the statistical analysis section.	√	
*4*	State the values used for statistical parameters (such as significance level and power).	√	√
*5*	State the underlying basis for determining the target difference:	√	
	a. an important difference as judged by a stakeholder,		
	b. a realistic difference based upon current knowledge or		
	c. both an important and realistic difference.		
*6*	Express the target difference according to the outcome type:	√	√
	a. Binary: state the target difference as an absolute and/or relative effect, along with the intervention and control group proportions. If both an absolute and a relative difference are provided, clarify if either takes primacy in terms of the sample size calculation.		
	b. Continuous: state the target mean difference on the natural scale, the common SD and standardised effect size (mean difference/SD). It is preferable to also provide the anticipated control group mean even though it is not required for the sample size calculation.		
	c. Time-to-event (survival): state the target difference as an absolute and/or relative difference, provide the control group event proportion, along with the intervention and control group survival distributions. Additionally, the planned length of follow-up should be stated along with the assumed accrual pattern. If both an absolute and a relative difference are provided, clarify if either takes primacy in terms of the sample size calculation.		
*7*	Explain the choice of target difference: specify and reference any formal method used or relevant previous research.	√	
*8*	State the sample size based upon the assumptions specified above (for a time-to-event outcome, the number of events required should also be stated). If any factors are incorporated which alter the required sample size (such as allowance for loss-to-follow-up) they should also be specified along with the final sample size.	√	√
*9*	Reference the trial protocol		√

**Table 3:** **Reworked example RCT protocol sample size calculation sections**

**Primary outcome type**	**Example text**
*Binary*	Men After Prostate Surgery (MAPS) radical prostatectomy trial [28]: The primary outcome is continence. The sample size was based upon a target difference of 15% absolute difference (85 versus 70%). This magnitude of a target difference was determined as both a realistic and important difference from discussion between clinicians and the project management group, and inspection of the proportion of urinary incontinence in the trials included in the Cochrane systematic review [29]. The control group proportion is also based upon the observed proportion in the RCTs in this review. Setting the statistical significance to the two-sided 5% level and seeking 90% power, 174 participants per group are required; 348 in total.
*Continuous*	Full-thickness macular hole and Internal Limiting Membrane peeling Study (FILMS) [7]: The primary outcome is ETDRS (Early Treatment Diabetic Retinopathy Study) distance visual acuity. A target difference of a mean difference of five letters, with a common standard deviation (SD) of 12, was assumed as five letters is equivalent to one line on a visual acuity chart and is viewed as an important difference by patients and clinicians. The SD value was based upon two previous studies; one RCT and one observational comparative study [30,31]. This target difference is equivalent to a standardised effect size of 0.42. Setting the statistical significance to the two-sided 5% level and seeking 90% power, 123 participants per group are required; 246 in total.
*Time to event*	Arterial Revascularisation Trial (ART) [32]: The primary outcome is all-cause mortality. The sample size was based upon a target difference of 5% in 10-year mortality with a control group mortality of 25%. Both the difference and control group mortality proportions are realistic based upon a systematic review of observational (cohort) studies [33]. Setting the statistical significance to the two-sided 5% level and seeking 90% power, 1464 participants per group are required; 2,928 participants (651 events) in total. Participants will be followed for 10 years.

## Discussion

The RCT is widely considered to be the best method for comparing the effectiveness of health interventions [1]. Determining the target difference is a key element of an RCT design. Improved standards in both RCT sample size calculations and reporting of these calculations would aid health professionals, patients, researchers and funders in judging the strength of the available evidence and would ensure better use of scarce resources. While no single method provides a perfect solution to a difficult question, we have provided practical guidance for researchers on sample size calculation with reference to specifying the target difference and how this should be reported in trial protocols and reports. To our knowledge, no alternative guidance exists. Although our examples and framing are from a medical context, the issues are relevant to social care, animal and other non-medical research as well. Further research into the implementation, practicality and consequence of using alternative methods for specifying the target difference (such as health economic and opinion-seeking), and exploration of the justification of some methods (such as the standardised effect size method, where the magnitude of the effect is used to infer the important of a difference) is needed.

## Conclusions

Specification of the target difference for the primary outcome is a key component of an RCT sample size calculation. There is a need for better justification of the target difference and for corresponding reporting of its specification. Raising the standard of RCT sample size calculations would aid health professionals, patients, researchers and funders in judging the strength of the evidence and would ensure better use of scarce resources.

## Abbreviations

ART: Arterial Revascularisation Trial
CONSORT: Consolidated Standards of Reporting Trials
DELTA: Difference Elicitation in TriAls
ETDRS: Early Treatment Diabetic Retinopathy Study
FILMS: Full-thickness macular hole and Internal Limiting Membrane peeling Study
MAPS: Men After Prostate Surgery
MCID: Minimal(ly) clinical(ly) important difference
MRC: Medical Research Council
NIHR: National Institute for Health Research
RCT: Randomised controlled trial
SD: standard deviation
SPIRIT: Standard Protocol Items: Recommendations for Interventional Trials
